# Improvement of shared decision making in integrated stroke care: a before and after evaluation using a questionnaire survey

**DOI:** 10.1186/s12913-019-4761-2

**Published:** 2019-12-05

**Authors:** H. R. Voogdt-Pruis, T. Ras, L. van der Dussen, S. Benjaminsen, P. H. Goossens, I. Raats, G. Boss, E. F. M. van Hoef, M. Lindhout, M. R. S. Tjon-A-Tsien, H. J. M. Vrijhoef

**Affiliations:** 1 Stroke Knowledge Network Netherlands, Oudlaan 4, 3515 Utrecht, GA Netherlands; 2 EnCorps, Goudenregenlaan, 16 1214 ND Hilversum, Netherlands; 3 Netherlands Society of Rehabilitation Medicine, Oudlaan 4, 3515 Utrecht, GA Netherlands; 4 Maastricht University Medical Center, P. Debyelaan 25, 6229 Maastricht, HX Netherlands; 5 Branch Organization of Rehabilitation in the Netherlands, Oudlaan 4, 3515 Utrecht, GA Netherlands; 6 Patient Association for Acquired Brain Injury, Den Heuvel 62, 6881 Velp, VE Netherlands; 7 Dutch General Practitioners’ Expert Group on Cardiovascular Diseases, Mercatorlaan, 1200 3528 Utrecht, BL Netherlands; 8 Panaxea, Science park 400, 1098 XH Amsterdam, Netherlands

**Keywords:** Shared decision making, Stroke, Rehabilitation, Implementation science, Integrated health care systems

## Abstract

**Background:**

Shared decision making (SDM) is at the core of policy measures for making healthcare person-centred. However, the context-sensitive nature of the challenges in integrated stroke care calls for research to facilitate its implementation. This before and after evaluation study identifies factors for implementation and concludes with key recommendations for adoption.

**Methods:**

Data were collected at the start and end of an implementation programme in five stroke services (December 2017 to July 2018). The SDM implementation programme consisted of training for healthcare professionals (HCPs), tailored support, development of decision aids and a social map of local stroke care. Participating HCPs were included in the evaluation study: A questionnaire was sent to 25 HCPs at baseline, followed by 11 in-depth interviews. Data analysis was based on theoretical models for implementation and 51 statements were formulated as a result. Finally, all HCPs were asked to validate and to quantify these statements and to formulate recommendations for further adoption.

**Results:**

The majority of respondents said that training of all HCPs is essential*.* Feedback on consultation and peer observation are considered to help improve performance. In addition, HCPs stated that SDM should also be embedded in multidisciplinary meetings, whereas implementation in the organisation could be facilitated by appointed ambassadors. Time was not seen as an inhibiting factor. According to HCPs, negotiating patients’ treatment decisions improves adherence to therapy. Despite possible cognitive or communications issues, all are convinced patients with stroke can be involved in a SDM-process. Relatives play an important role too in the further adoption of SDM. HCPs provided eight recommendations for adoption of SDM in integrated stroke care.

**Conclusions:**

HCPs in our study indicated it is feasible to implement SDM in integrated stroke care and several well-known implementation activities could improve SDM in stroke care. Special attention should be given to the following activities: (1) the appointment of knowledge brokers, (2) agreements between HCPs on roles and responsibilities for specific decision points in the integrated stroke care chain and (3) the timely investigation of patient’s preferences in the care process – preferably before starting treatment through discussions in a multidisciplinary meeting.

## Background

The importance of shared decision making (SDM) is becoming recognised, not only for ethical reasons and respecting the autonomy of the patient, but also for balancing the benefits of treatment against the risks, costs and harm [1]. SDM has been defined as: ‘an approach where clinicians and patients share the best available evidence when faced with the task of making decisions, and where patients are supported to consider options, to achieve informed preferences” [2]. Research has underlined the positive outcomes of SDM in terms of benefits for patients, including improved understanding, satisfaction, trust, treatment adherence and health outcomes [3]. In addition, SDM can result in benefits for the healthcare system such as improved satisfaction among HCPs and optimum resource utilisation [4]. Nevertheless, the evidence for the effects of SDM interventions is still inconclusive because the certainty of the evidence is low or very low [5] and further research on implementation strategies is needed. Multifaceted implementation strategies among HCPs, their organisations and patients to deal with barriers and facilitators for change can improve SDM in clinical practice. Examples of barriers to practising SDM in clinical settings include negative attitudes towards SDM, lack of familiarity with SDM, insufficient explanations during consultation and a lack of resources [6, 7]. Facilitators of SDM include motivated HCPs, the perceived benefits of SDM, adequate consultation time, engagement of various team members [4, 8]. Implementation programmes for SDM include several activities assisting the above-mentioned facilitators. Examples are tapping into motivations to engage with SDM, providing training with role play, aiming for quality improvement and monitoring outcomes, using local facilitators, using SDM tools that are tailored to the setting, creating mapping tools to help understand how care pathways can support SDM and where the decision points are [5].

Although SDM has proved to be beneficial in terms of health outcomes when implemented under controlled conditions, practising SDM could become complex in integrated stroke care [9–11]. After a stroke, patients receive integrated care in collaborative networks of healthcare and social care providers. In the Netherlands, integrated stroke care is organised in stroke services; regional networks of providers working together during the acute, rehabilitation and chronic phases of stroke care. Stroke services aim to deliver coherent and patient-centred, integrated care. About 70% of patients are discharged from hospital back home; about 20% are referred to rehabilitation centres or nursing homes. Patients face several decision points about treatment options and the setting in which care takes place [12, 13]. In addition, strokes can limit patients’ understanding of complex information about care options and their anticipated outcomes, consequently impeding patient participation in the decision-making process [14]. Cognitive problems in patients with stroke (such as memory problems, a poor understanding of the condition or the inability to judge adequately) may hamper the SDM process. Communication problems such as aphasia or dysarthria could also hinder the SDM process. An implementation programme for SDM was therefore started in five Dutch stroke services.

## Methods

### Aim

This before and after evaluation study aimed to identify factors for implementation of SDM in integrated stroke care and to conclude with key recommendations for further adoption of SDM in Dutch stroke care. Specific research questions were: According to HCPs, is SDM feasible for patients that suffer from stroke? What factors influence the implementation of SDM? What does this mean for the further embedding of SDM in Dutch integrated stroke care?

### Study design

A one-year SDM implementation programme was carried out in five stroke services. A before and after evaluation study was used to obtain a clear picture of the facilitators and barriers to the implementation of SDM in stroke rehabilitation care. A baseline self-administered e-mail questionnaire to all participating HCPs at five stroke services in the programme was followed by in-depth interviews. Most of the participating HCPs worked in the (out-patient) clinic of a rehabilitation centre and/or primary care. After six months, at the end of the programme, a second e-mail questionnaire to all participating HCPs was used to validate and quantify interview statements.

### Implementation programme

All 75 Dutch stroke services were invited to participate in the SDM implementation programme (Table 1). Five stroke services responded. Criteria for participation in the programme were involvement in the regional team of both primary and secondary HCPs and the willingness/ability to implement SDM. In the preparation stage of the implementation programme (July–November 2017), all regional teams received SDM training (online training of three hours and three hours of practical training in SDM). The e-learning consisted of theory about the conceptual framework of SDM, the importance and advantages of SDM, reflection on current practice and the steps of SDM in a consultation as described in literature. The skills training sessions consisted of reflection and discussions about barriers and facilitators for implementing SDM, practical instructions for engaging and assisting patients in decision making, and practising skills for SDM through role playing. In addition, each regional team created an action plan for implementing SDM in their daily care practice, focusing on three self-chosen SDM specific topics in stroke care (for example, what is the best option for speech and language therapy for follow-up stroke care for a specific patient: the outpatient rehab clinic, aphasia centre or home based care?). Furthermore, HCPs received a pocket card with the steps of SDM and example statements and sentences as a quick reference guide. The five regional teams first started implementing SDM in their consultations in December 2017. With help from the project team, they developed tools for assisting the SDM process for with patients with stroke, such as decision aids for each of the three chosen decision points in the care chains, including details about the available options, the advantages and disadvantages for each of these options and which preferences, concerns and expectations may be important for patients. The implementation activities of the regional teams were guided by the project team; they received personal feedback, instructions and training on integrating the SDM process (audio recordings of consultations were scored by the validated Option5 instrument [15] and feedback was presented to the individual HCP in order to improve their SDM skills) in their consultations and organisation. The project team’s guidance and coaching of the participants consisted of regular consultations with the local team leader to monitor progress on the action plans (approximately once every two weeks), attending team meetings and organising SDM training sessions. After the implementation, programme teams organised open meetings for stroke care professionals in their region to share experiences about SDM and to further spread the concept of SDM in the care chain.

**Table 1:** Overview Implementation programme

INTERVENTIONS	MAIN AIMS	PERIOD
Start-up meeting (local team)	- Overview programme	Apr’17
Meeting (local team)	- Assignment team	June-Aug‘17
Make-up of local implementation action plan (local team)	- Decision points	July-Sep‘17
First national meeting (with 5 teams)	- Overview programme - Theory on SDM - Learning from each other	18 Sep‘17
Launch of e-learning on SDM	- Knowledge of SDM	Sep‘17
Personal feedback on recordings of consultation (at least one consultation for each team-member) – by mail	- Personal skills	Sep-Oct‘17
Basic training on SDM (local team + colleagues) (experienced trainers)	- Learning from each other - Personal skills	Oct-Nov‘17
Follow-up meetings (local team)	- Defining local action plan	Sep-Dec‘17
Baseline measurement		
Meeting with 5 team-leaders	- Decision tools - Implementation activities	Dec‘17
Commencement of SDM into clinical practice (all teams)	- Implementation activities	Dec’17
Second national meeting (with 5 teams)	- “All teach, all learn” - Implementation activities	Jan‘18
Follow–up meetings (local team)	- Decision tools - Implementation activities	Feb-Apr‘18
Follow-up training on SDM (local team + colleagues) (experienced trainers)	- Learning from each other - Personal skills - Implementation activities	Mar-Apr ‘18
Final measurement		
Final meetings in 5 stroke services (spread of ‘lessons learnt’) (local team + colleagues)	- “All teach, all learn” - Dissemination activities	June–July‘18 (* one team in Nov‘18)

### Study population

All 25 HCPs of five stroke services who participated in the regional project teams were approached for a baseline e-mail questionnaire. Subsequently, 11 HCPs were selected for in-depth interviews; they represented all participating regions and professional disciplines (rehabilitation nurse, occupational therapist, physiotherapist, speech therapist, psychologist, rehabilitation specialist and care manager). Data saturation was expected with 10–12 interviews. At the end of the implementation programme, all participating HCPs were approached again for a final measurement.

### Questionnaires and data collection

Two methods of data collection were used for the baseline measurement. The self-administered e-mail questionnaire (December 2017) aimed to obtain a clear picture of the initial views and expectations of team members about SDM and its future implementation in stroke services. All 25 HCPs received an open-ended questionnaire with five questions. This questionnaire addressed the four domains of the model of ‘determinants of the innovation in health care organisations’ (MIDI-model) by Fleuren et al. [16]: (1) the innovation, in this case “SDM”, (2) the user, in this case “patient” and “care provider”, (3) the preconditions regarding organisation and (4) the preconditions regarding the system. This questionnaire was drawn up by three authors (HRVP, TR, HJMV) (Additional file 1).

The later in-depth interviews (January 2018) aimed to obtain further details about the initial e-mail questionnaire results. Therefore a topic list drawn up from the initial e-mail questionnaire results by three authors (HRVP, TR, HJMV) was used for the interviews. Topics were assigned based on all sub-themes of the four domains of the MIDI model by Fleuren et al. [16] and the additional subthemes for the two domains ‘organization’ and ‘system’ from the scoping review by Scholl et al. [17] concerning the organizational- and system level characteristics that are likely to influence the implementation of SDM. The results of the baseline measurement were shared at the local team meetings, but no subsequent implementation actions were formulated at that time. At the end of June 2018, all HCPs were approached for a final measurement using a questionnaire to verify and quantify facilitators and barriers for implementation of SDM. This questionnaire contained 51 statements, derived from a qualitative analysis of the in-depth interviews at baseline (data analysis was done by HRVP, TR, HJMV). In this questionnaire, a five-point scale of response categories (ranging from entirely disagreeing to entirely agreeing) was used. For each statement, participants could state how much they thought it was “essentially important for the implementation of SDM”. If a statement was considered essentially important for implementation, participants were asked what actions (opportunities/solutions) they thought essential for tackling the factor in question. The face validity of the questionnaire was tested by team members (GB, IR, LvdD, PHG, MRST). The questionnaire at baseline was sent by e-mail. The final questionnaire was sent using Google Forms. The in-depth interviews at baseline were conducted by an independent interviewer (TR) who was not involved in the implementation programme and did not know the participants. Audio recordings were made with the permission of the interviewees and informed consent was received. The interviews lasted around 45 min.

### Data analysis

#### Qualitative analysis

The recordings of the in-depth interviews at baseline were transcribed verbatim. The transcripts were encoded by three researchers (HRVP, TR, HJMV), using two coders for each interview. The encoding was based on determinants mentioned in the MIDI model by Fleuren et al. [16] and the scoping review by Scholl et al. [17]. All results were deductively coded to the domains and sub-themes concerning the determinants. In addition, the analysed data were used for the development of the questionnaire at final measurement.

#### Quantitative analysis

Data at the final measurement were analysed using SPSS 17.0 and frequencies were calculated. Key factors for implementation of SDM in stroke care were selected by a high level of agreement with the statement (≥ 4.0) and a high percentage of importance (≥ 75%) and are illustrated by a quote (shown in italics). Factors needing no further attention for implementation were excluded by a low level of agreement with the statement (≤ 2.0) and a low percentage of importance (≤ 25%). Results were prioritised by (1) the extent to which respondents agreed with the statement in question and (2) the extent to which the aspects mentioned in the statement were deemed essentially important for the implementation of SDM. The actions (opportunities/solutions) that HCPs deemed essential for implementation were summarised and transformed into key recommendations for further implementation.

## Results

### Respondents

22 HCPs (88% of 25 HCPs) responded once in this evaluation study. 21 HCPs responded to the baseline mail questionnaire and 16 HCPs to the questionnaire at the end of the programme. In-depth interviews were held with 11 HCPs (Table 2). All professional disciplines involved in the programme responded to the final questionnaire.

**Table 2:** Study population and participation in evaluation study (number of HCPs)

Type of professional	In regional teams	Baseline questionnaire	In-depth interview	Final questionnaire
Rehabilitation nurse	5	4	2	3
Occupational therapist	3	3	1	2
Physiotherapist	5	5	2	3
Speech therapist	2	2	1	1
Psychologist	4	2	1	1
Practice/home care nurse	2	2	0	2
Rehabilitation specialist	2	1	2	2
Care manager	2	2	2	2
**Total**	25	21	11	16

### Expectations and experience with SDM in integrated stroke care

#### The healthcare provider and the preconditions regarding the organisation

According to the majority of HCPs, *“it is important that all HCPs involved are trained in SDM”* (Table 3, statement 8). Training of HCPs is also essential for implementing SDM in stroke care. *“Some colleagues do not recognise the shortcomings in their consultations and training helps them to become more aware of them.”* “*The theory of SDM encompasses a simple set of concepts, but it really takes some effort to put it into practice.”* Feedback on consultation audio recordings (Table 3, statement 12) helps HCPs to reflect on their performance. *“You have to make audio recordings to become aware of your own skills”, “as they recognise their incompetence, they consciously acquire a skill [ …*] *eventually, the skill can be utilised without it being consciously thought through”* (Table 3, statement 13). In addition, HCPs stated that regularly reflection with colleagues on how consultations are conducted is needed (Table 3, statement 13). *“It would be nice if peer observation among colleagues became more common … that’s quite a challenge; some are reluctant to do so.*” For further implementation of SDM, all colleagues involved should be convinced of the added value of SDM for stroke care (Table 3, statements 2 and 3) *“Show the possibilities and added value of SDM, particularly to rehabilitation specialists who are used to fast decision-making about treatment [...]”* The process of SDM should also be embedded in the multidisciplinary meetings; it is important to take all “preferences, wishes and worries” of patients into consideration when discussing follow-up care (Table 3, statement 5). “*A case manager could collect the preferences and wishes beforehand...”* and other roles and responsibilities in the SDM process should be explicitly shared among colleagues (Table 3, statement 1) *“In personal health records, I now add the outcomes of the SDM process to make this clear for my colleagues.”* For improving SDM in decisions on follow-up care, HCPs in the rehabilitation centre need to have information about the primary HCPs available (Table 3, statement 6). Remarkably, HCPs did not state that time was an inhibiting factor in the implementation, after finalising the programme, whereas this was mentioned several times at the start of the programme *“If you handle SDM properly, aims for treatment become more clear [ …*] *finish sooner”, “the current way of explaining the treatment options to patients is sometimes messy … in particular when more colleagues are involved”, “To be honest, I don’t expect it to take more time [ …*] *so far, we haven’t planned more consultations in order to make a shared decision.”* (Table 3, statement 36).

**Table 3:** Level of agreement with statement and % of HCPs deeming the factor essential for the implementation of SDM in stroke care

STATEMENT		Entirely disagree (% HCPs)	Disagree (% HCPs)	Disagree/agree (% HCPs)	Agree (% HCPs)	Entirely agree (% HCPs)	Average level of agreement (% HCPs)	Essential implementation factor	HCP recommendations
**No**		1	2	3	4	5		%	
**The care provider and the preconditions regarding the organisation**									
	**Strong agreement (≥ 4.0) & essential for SDM implementation (≥ 75%)**								
**8**	If SDM is to be implemented properly, all the healthcare professionals involved must be trained in it.	0	7	13	53	27	4.0	80	• e-learning • in-company training • regional training (a meeting place) • use of tools for making choices
**5**	To promote the implementation of SDM, the “preferences, wishes and worries” of the patients must always be covered when discussing the follow-up treatment at the multidisciplinary meeting.	0	0	33	20	47	4.1	80	• a broad approach, not only stroke • good preparation for the multidisciplinary meeting: fixed point of contact to request preferences • in a knowledge broker project
	**Less strong agreement (< 4.0),** **however essential for SDM implementation (≥ 75%)**								
**2**	I need to convince my colleagues that SDM has added value. Quote: “If you can show a few examples, it’ll help me get my colleagues on board.”	0	7	13	73	7	3.8	80	• collect examples/case studies • in-company training • in an electronic medical record
**6**	Secondary healthcare professionals find it difficult to get a clear picture of the quality and content of what is available for stroke patients in primary care. Who has the expertise? What care is provided, exactly? What are the potential pros and cons for the patient?	7	27	27	33	7	3.1	80	• ‘work charts’ with the care options • overview of the expertise available within the care chain (social map)
	**Strong agreement (≥ 4.0), however less essential**								
**12**	Listening to an audio recording of a consultation lets me reflect upon how I conduct my conversations for SDM and improve it.	0	0	13	53	33	4.2	60	• in training
**1**	I need to agree with my colleagues who is doing what in the SDM process.	0	7	20	40	33	4.0	67	• discuss the division of roles for SDM • create ‘work charts’ in the care chain meeting or other meetings • reporting • peer review
	**Less strong agreement (< 4.0) and less essential (< 75%)**								
**7**	In SDM, transfer to another care provider must also include communication about going through the SDM process and its results.	0	7	13	60	20	3.9	73	• digital decision-making tools
**9**	SDM means that you as the care organisation provide less care that is unnecessary or redundant. Quote: “If the patient has made the right choice, they’re much more likely to stick to the therapy, so we have fewer no-shows.”	0	0	33	47	20	3.9	60	• added value of SDM in the media • in quality standards • explanation of ‘time investment’ • encouragement via health insurers
**4**	If SDM is to be implemented properly as the patient’s care progresses, all the healthcare professionals involved must be trained in it.	0	0	27	67	7	3.8	73	• reflection with healthcare professionals • setting up decision-making tools • via the professionals
**16**	If the patient chooses an option that I think is less good, I will still assist them. I think it is important to give the patients room. Quote: “Someone can make what I think is the ‘wrong’ decision, but still be perfectly happy with it.”	7	7	20	33	33	3.8	73	• state in the electronic medical records
**13**	For the implementation of SDM, I need to reflect regularly with my colleagues on how we conduct consultations.	0	7	33	47	13	3.7	60	• in peer review
**11**	An internal ambassador is needed for implementing SDM in my organisation.	7	13	20	40	20	3.5	53	• implementation on a project basis
**10**	For SDM in the stroke care chain, the healthcare professionals have to know each other personally. Quote: “If you know who you are referring people to, it all goes much more smoothly.”	0	13	40	40	7	3.4	40	• joint training or meetings • make agreements in regional networks
**3**	My colleagues assume incorrectly that they are already using SDM: they are ‘unconsciously incompetent’.	7	13	47	33	0	3.1	53	• put on the media’s agenda • use role models • reflect upon specific cases together
**14**	It is taking me longer than I thought to implement SDM in practice.	7	13	47	27	7	3.1	47	• explanation of time
**17**	Stroke patients sometimes have a limited picture of their condition. If they make a ‘wrong’ decision, I’ll try to block it. That’s my responsibility as a care provider.	13	20	33	33	0	2.9	73	• communicate better • good explanations for patients and their relatives • involve the relatives
**18**	My colleagues don’t have enough time to implement SDM properly in their practices.	13	20	40	20	7	2.9	40	• raise the issue if necessary with health insurers
**15**	I think that I don’t have enough time to implement SDM properly in my practice.	27	20	33	13	7	2.5	27	• good planning • making the roles clear
**System-related factors**									
	**Strong agreement (≥ 4.0) & essential for SDM implementation (≥ 75%)**								
**19**	For the further implementation of SDM, healthcare professionals need to make time free to practice and apply SDM.	0	0	20	60	20	4.0	80	• start-up an internal improvement project • arrange it throughout the organisation • on-the-job coaching
	**Less strong agreement (< 4.0), however essential for SDM implementation (≥ 75%)**								
**26**	The management must create the conditions for the staff to be able to learn SDM and implement it more broadly.	0	7	14	57	21	3.9	86	• in the organisation’s mission • in regular meetings • appoint an ambassador • facilitate training and time for exercises • demonstrate the added value to staff
	**Statement of low concern; low level of agreement/disagreement of HCPs and non-essential (≤ 2.0 and/or ≤ 25%)**								
**21**	The new privacy rules are obstructing SDM because they limit the options for exchanging information.	21	50	29	0	0	2.1	14	• patient/relative as the data manager
**24**	The care funding model means that healthcare professionals feel forced to be creative with the remuneration rules for SDM. For example, formulating an extra goal because a treatment in the rehabilitation centre would otherwise have to be discontinued. Or formulating an indication for taxi transport differently so that it will be remunerated.	21	21	36	14	7	2.6	14	
**22**	The culture in my organisation is obstructing the implementation of SDM. Quote: “… because there is in fact a culture of ‘it’s my way or the highway’.”	21	43	36	0	0	2.1	21	• training
**25**	High staff turnover of the healthcare professionals I work with is obstructing my efforts to apply SDM.	21	43	36	0	0	2.1	21	• good agreements • work using the same methods
	**Other**								
**20**	Documenting the process of SDM in the electronic health records lets you account for any deviations from a care protocol.	0	7	27	33	33	3.9	53	• make agreements
**23**	Care insurers must also shoulder their share of the responsibility for implementing SDM. They can for instance take a look at their purchasing processes to see what factors inhibit and encourage SDM in the stroke care chain.	7	21	21	29	21	3.4	36	• in discussions with health insurers
**27**	Patients who are discharged from the rehabilitation centre to primary care are incorrectly restricted in their freedom to choose ‘care closer to home’ by the funding system.	14	0	64	14	7	3.0	29	• social maps/work charts • avoid incorrect bed occupancy
**SDM as innovation**									
	**Strong agreement (≥ 4.0) & essential for SDM implementation (≥ 75%)**								
**33**	If I am to apply SDM, it is important that I use understandable language.	7	0	7	29	57	4.3	79	• be aware of the jargon • insights into health skills • treatment plan and decision-making tools in understandable language for the patient/relative • learn to communicate and practice it • use teach-back methods
**34**	If I am to apply SDM, it is important to use teach-back methods.	0	7	7	57	29	4.1	79	• awareness • in training
**38**	SDM makes the intrinsic motivations of the patient clearer.	0	7	7	57	29	4.1	86	• take time
	**Less strong agreement (< 4.0), however essential for SDM implementation (≥ 75%)**								
**31**	I think presenting the explanation of the treatment options neutrally is complex if I do not think they are fully equivalent.	7	36	43	7	7	2.7	86	• strength of your own expertise • acceptance of other choices • transparency about the evidence • simple working charts showing the features instead of the pros and cons • practice, get feedback, listen to recordings of conversations, reflection
	**Strong agreement (≥ 4.0), however less essential**								
**28**	The core of SDM is allowing the treatment to focus on the patient’s objectives more. Quote: “If you’ve got a clear picture of what the patient wants, you have a better idea of what possible treatments there are.”	0	0	14	64	21	4.1	64	• embed in the RAPP methodology • in training: learn to set objectives • listen properly to the patient
	**Statement of low concern; low level of agreement/disagreement of HCPs and non-essential (≤ 2.0 and/or ≤ 25%)**								
**30**	I find it difficult to discuss the role the patient can take in the SDM process with them. Quote: “I don’t yet really know how I can discuss the role the patient can take or would like to take with them.”	43	36	21	0	0	1.8	50	• training (on the job) • open attitude towards the patient
**39**	SDM is not a new method – we were doing it implicitly anyway; there is just a theoretical framework for it now.	0	14	57	21	7	3.2	21	• awareness of the paternalistic method • self-reflection • quality of life as the starting point instead of the limitations
	**Other**								
**37**	SDM leads to improved therapy compliance. “If this means a patient gets special shoes made, they’re really going to wear them.”	0	7	36	36	21	3.7	57	• express the message (increased therapy compliance and explanation of the alternatives) • be aware of changes that the patients themselves show – let them take more actions themselves for trying out the options, etc.
**35**	SDM makes the discussion is more pleasant.	0	0	50	29	21	3.7	57	
**40**	Applying SDM is turning out to be more awkward than I thought in the first instance. Now that I’m aware of what I’m doing, I can see that I’m not always applying all five steps properly. I’ve become ‘consciously incompetent’.	0	14	43	29	14	3.4	57	• in training
**36**	SDM seems to cost a lot more time, but I have not needed any extra consultations for taking decisions together with the patient.	14	21	29	21	14	3.0	50	• express the message • agree within the team who will work out the details of the options. Preliminary work done by the contact
**32**	SDM is costing me too much time. Quote: “You really do have to sit down properly with the patient for it. It takes a lot of time.”	21	21	43	7	7	2.6	57	• explanation of time (therapy compliance) • the management provides room
**29**	You have to learn the points in the care process for an individual patient at which you should use SDM. Quote: “SDM is tricky in practice: what situations are there where a decision could depend on preferences?”	14	29	43	7	7	2.6	57	• awareness • training and reflection
**Factors related to the patient and their relatives**									
	**Strong agreement (≥ 4.0) & essential for SDM implementation (≥ 75%)**								
**49**	A stroke patient’s relatives play a major role in SDM.	0	7	21	36	36	4.0	79	• always involve the relatives • evenings for informal caregivers
	**Statement of low concern; low level of agreement/disagreement of HCPs and non-essential (≤ 2.0 and/or ≤ 25%)**								
**45**	Stroke patients don’t want to be involved in the decision because they don’t want to take any responsibility.	64	0	29	7	0	1.8	29	• a principle for care
**41**	The acute phase of stroke care is not really suitable for SDM.	14	36	21	21	7	2.7	21	• see what is possible for each situation
	**Other**								
**43**	The implementation of SDM must in fact also be driven by the patients and their relatives (bottom up). They must ask for SDM from the healthcare professionals. For instance, the patient or relatives must spontaneously ask the care provider about other options and the pros and cons of those options, or they must state their own values so that a good decision can be made.	0	7	21	50	21	3.9	57	• via patients’ organisations • use decision-making tools • information about SDM
**50**	SDM puts the lives of patients more at the centre.	0	0	36	36	29	3.9	50	• focus on the quality of life
**48**	In the chronic phase, almost all stroke patients can help decide about the care.	7	0	36	21	36	3.8	50	• informal caregiving is always a possibility
**42**	SDM is difficult if I have doubts about the mental competence of the patient.	7	14	21	29	29	3.6	43	• involve a relative • peer review
**51**	The implementation of SDM must in fact also be driven by the patients and their relatives. They must explicitly start choosing care organisations or care professionals who provide scope for SDM.	0	14	43	29	14	3.4	43	• pay attention in the media
**44**	I think it is easy to estimate what role the patient will be able to take in SDM throughout the recovery process. Quote: “Sometimes it starts with very small choices: do you want a raspberry drink or a lemon drink... you can get the patient used to taking decisions again.”	14	7	29	29	21	3.4	36	• be aware that the patient can always have a different role in SDM • let people take control as much as possible
**47**	Stroke patients are inhibited by their brain damage: cognitive problems mean that they can’t assist in decision-making.	29	14	50	7	0	2.4	57	• keep looking for the possibilities for each patient • involve the relatives
**46**	Stroke patients don’t want to join in the decision-making because they think the healthcare professionals – such as me – know what’s best.	43	14	29	14	0	2.1	36	• a care provider must support the patient in this regard

#### System-related factors

People need to train their SDM skills and try to put SDM into practice (Table 3, statement 19). Managers should assist this for all employees in their organisations (Table 3, statement 26). The new privacy legislation, the funding system, the culture within the organisation and the high turnover of colleagues (Table 3, statements 21, 24, 22 and 25) are factors that were mentioned at the start of the project but ultimately seemed to be less essential for further implementation of SDM. *“In May 2018, the European privacy legislation on sharing personal healthcare data became stricter [...] maybe it will be an obstacle in the long term [ …*]”.

#### SDM as innovation

According to HCPs, SDM improves patient-centred care (Table 3, statement 28) “*If HCPs know the aims and wishes of the patients [...] it makes it easier to choose between treatment options.”* And *“it also clarifies the intrinsic motivations of the patient”* (Table 3, statement 38). In opinion of HCPs, negotiating patients’ treatment decisions improves adherence to therapy (Table 3, statement 37). *“If the patient makes the right choice, they are more likely to stick to the therapy [ …*] *there will be fewer no-shows [ …*] *orthopaedic shoes will be worn”.* SDM requires clear and understandable communication and the use of the teach-back method (Table 3, statements 33 and 34). HCPs sometimes experience difficulty presenting information on treatment methods available neutrally, especially when the treatment options are complex.

#### Factors related to the patient and their relatives

Despite possible cognitive or communications issues, HCPs are convinced patients with stroke can be involved in SDM (Table 3, statements 45 and 47). *“It is not easy, though … sometimes it is hard to assess a patient’s cognitive skills*.” Relatives therefore play an important role in the process of SDM (Table 3, statement 49). SDM should be promoted to patients and their relatives by patients’ organisations (Table 3, statement 43) specifically for patients receiving stroke rehabilitation or chronic stroke care (Table 3, statements 41 and 48).

### Recommendations

HCPs gave several practical recommendations for further adoption of SDM in integrated stroke care (Table 3). These recommendations have been summarised and transformed into eight practical recommendations for further adoption of SDM in stroke services (Table 4).

**Table 4:** Practical recommendations for further adoption of SDM in stroke care according to HCPs

1. Start an awareness campaign	Promotional material; SDM in strategic plans; articles on best practices and experiences with SDM (by well-known HCPs); reports on benefits; tools for self-reflection on SDM.
2. Appoint ambassadors in the organisation	Activities to improve SDM in daily practice; involvement of staff and all HCPs.
3. Include SDM in training and courses of HCPs	In courses for HCPs. Topics: relational communication, neutral communication, decision-making dilemmas, teach-back. Include role play, personal reflection and peer observation.
4. Training on the job	E-learning courses and in-company training to practice and develop skills. Topics: relational communication, neutral communication, decision-making dilemmas, teach-back. Include role play, personal reflection and peer observation.
5. What matters to patients?	Investigation of personal preferences before starting treatment discussions in multidisciplinary meeting; Personal decision tools to state preferences, documentation in electronic health record.
6. Involve relatives	Proper involvement of relatives because of cognitive and communication problems that can occur. Explanations of roles and responsibilities.
7. Implementation via stroke services and care chains	In meetings of regional and organisational team: discuss how to embed SDM in existing care chains. Agreements on roles and responsibilities for specific decision points for example “transfer charts”.
8. Provide an overview of the available primary care for strokes in the region	An informative overview of primary HCPs and organisations who have expertise in stroke care to assist SDM on transfer to primary care. Develop/include quality indicators for primary care in terms of volume norms and requisite training

## Discussion

According to HCPs, training of all HCPs, including personal feedback on consultation and peer observation, is essential for the implementation of SDM in integrated stroke care*.* The importance of training and personal feedback is also presented in earlier studies [5, 6, 18]. Contrary to these studies, ‘time’ was not regarded as an inhibiting factor. As HCPs in integrated stroke care felt that *“their current way of explaining the treatment options to patients were sometimes messy or time-consuming when more professionals are involved”*, and as most of them do not have a comprehensive overview of all the options available for stroke care in primary care, time could be saved by using decision aids and mutually agreeing roles and task in the SDM process [18, 19]. HCPs also emphasised the importance of embedding SDM in multidisciplinary meetings. Implementation of SDM in the organisation could be facilitated by appointed ambassadors in the stroke services. They could help to improve its implementation, as a tailor-made intervention to deal with reported or observed barriers to change. As such, it may turn out to be more effective than interventions that are generic and not tailored to context-specific barriers [20] Ambassadors or knowledge brokers for SDM are essential, as no project team is available in clinical practice to promote SDM (which was the case in this implementation programme). Despite possible cognitive or communications issues, all HCPs are convinced patients with stroke can be involved in the SDM process. The communication methods used by HCPs and relatives turned out to reveal hidden competencies of patients with communication problems and to improve patient participation [21, 22]. The relatives, therefore, play an important role too.

Because of its focus on SDM implementation in stroke services, our study adds unique insights into implementing SDM in multidisciplinary care chains and a population of patients who may possibly have limited understanding because of their medical condition. This study is the first that presents barriers and facilitators for SDM with patients with stroke. The strength of this study is the pre-post mixed-method design. It let us validate the results from the first phase of this study and hence to draw robust conclusions. In addition, most if not all professional disciplines working in stroke care participated and the response rate was relatively high. In this study, actual experience with implementing SDM has been investigated. This may have minimised the risk of recall bias. The in-depth interviews in this study were held by an independent researcher who was not involved in the project team. This study, however, has also some weaknesses. Because participation in our study was partly voluntary and the participating HCPs are more likely to be motivated towards implementation of SDM (early adopters), our study population is probably not representative of the entire population of HCPs involved in stroke care. Although we used two models for the implementation of innovation [16, 17] to compile the topic list – and the response rate was high – it is possible our study has missed certain barriers and facilitators that would be expected and/or experienced by late adopters. Although patients were involved in the development and evaluation of decision aids, a significant shortcoming of this study is the absence of participation by patients or patient representatives. Further research on the experiences of patients and relatives with SDM in integrated stroke care is strongly recommended.

In accordance with an earlier study [23], accelerated implementation of SDM should preferably start bottom-up, within the local setting, by adapting the way HCPs conduct their consultations, adapting the cooperation between HCPs, patients and relatives, and by explicitly sharing responsibilities in the SDM process. All HCPs working together in a care chain or clinical pathway (e.g. for strokes) should be engaged in training for SDM (for instance on the job) – discussing common decision points - to make SDM common practice among stroke services. All the HCPs involved need to embrace and practise the common societal value of patient values and quality of life as the base for healthcare delivery with SDM as a common agent, not as the goal.

## Conclusion

Our study indicated it is feasible to implement SDM in integrated stroke care and several well-known implementation activities could improve SDM in stroke care. Still, some activities are specifically needed to address barriers and facilitators in integrated care for patients with stroke. Surprisingly, HCPs didn’t feel the time implications were as important after implementation as before. In addition, they gave several practical recommendations for further adoption of SDM in integrated stroke care. Three of the key recommendations for further adoption of SDM in stroke services, are remarkable: (1) To improve SDM in daily stroke care, stroke services should appoint ambassadors or knowledge brokers for multifaceted implementation strategies among HCPs, organisations and patients, in order to deal with barriers and facilitators for change. (2) Participating HCPs should discuss how to embed SDM in the integrated stroke care. Agreements on roles and responsibilities for specific decision points will improve SDM in stroke care. For all important decision points, decision tools with an overview of options - with possible benefits and risks should be developed. (3) Despite possible cognitive or communications issues, patients with stroke can be involved in SDM. Implementation strategies on SDM should aim to embed the timely investigation of patients preferences in the care process - before starting treatment discussions in multidisciplinary meeting.

## Supplementary information

**Additional file 1.** Baseline questionnaire and the domains (italicised) of the MIDI model [16].

## Data Availability

All transcripts of interviews and questionnaire results are archived by the first author (HRVP).

## Abbreviations

HCP: Healthcare professional
HCPs: Healthcare professionals
SDM: Shared decision making
