# Supplementary material for: Improvement of shared decision making in integrated stroke care: a before and after evaluation using a questionnaire survey
Source: BMC Health Serv Res. 2019 Dec 5;19:936. doi: 10.1186/s12913-019-4761-2 (PMC6896582; doi:10.1186/s12913-019-4761-2)
Supplement: Supplementary file 1 — Additional file 1. Baseline questionnaire and the domains (italicised) of the MIDI model [16]. [file 12913_2019_4761_MOESM1_ESM.docx]

**Additional file**

**Baseline questionnaire and the domains (italicised) of the MIDI model [16].**

1. Please state in your own words what you think of SDM. Consider aspects such as the theory, the added value of SDM, the complexity, how it fits in with practice, etc. *“The innovation”*
2. How do patients with stroke perceive and respond to SDM? What are your opinions about this? What positive experiences or obstacles do you see? *“The user (the patient)”*
3. What does the application of SDM mean for you as a care provider? *“The user (the care provider)”* (For example your role with respect to the patient, your responsibilities, your skills, holding consultations, conflicts with your opinions.)
4. What are the consequences of SDM for the care process and cooperation? (For example the division of roles between colleagues, treatment plan discussions, transmural cooperation, consultations, consultation times, etc.) *“The organisation”*
5. What aspects within the organisation or the preconditions affect how SDM is applied in the stroke care chain? (For example protocols, the agreements on transmural cooperation, and production norms, funding of care, legislation, primary/secondary care expertise, etc.) *“The context”*
